# Supplementary material for: Quantifying the benefit of a proteome reserve in fluctuating environments
Source: Nat Commun. 2017 Oct 31;8:1225. doi: 10.1038/s41467-017-01242-8 (PMC5663898; doi:10.1038/s41467-017-01242-8)
Supplement: Supplementary file 1 — Supplementary Information [file 41467_2017_1242_MOESM1_ESM.pdf]

Supplementary Note 1: Theoretical analysis of upshifts

We will describe in this note a kinetic model for upshifts, rooted in the work of several authors such as Schleif [1] and Dennis & Bremer [2, 3]. We will then link the kinetics to the protein allocation model introduced in [4]. The list of all symbols used can be found in Supplementary Table 1.

1.1 Kinetic modelling of the proteome composition

The proteome can be separated into different sectors ϕ, depending on the growth rate dependency of the individual proteins [4]. These sectors, defined as mass fractions, i.e. proteins of mass M_j have the mass fraction $\phi_{\rm j} = M_j/M$ of the total protein mass M, sum up to unity

$$\sum_j \phi_j(t) = 1. \tag{1}$$

The R-sector $\phi_{\rm R} = M_{\rm R}/M$ is the most prominent sector [4], and it mostly contain ribosomes and ribosome-affiliated proteins (e.g. elongation factors). It increases if growth rate increases due to better growth media. The R-sector $M_{\rm R}$ can be thought of as the mass of the "extended ribosomes", that is, ribosomal proteins plus their ribosome-affiliated proteins. The total mass of the R-sector proteins $M_{\rm R}$ is about 1.6 times larger than the mass of the ribosomes alone [5]. Due to the constraint of Eq. (1), an increase of the R-sector means a decrease of another sector.

Protein synthesis is proportional to the mass of R-sector proteins $M_{\rm R}$ times the "translational efficiency" $\sigma(t)$,

$$\frac{{\rm d}M(t)}{{\rm d}t} = \sigma(t) M_{\rm R}(t) \ , \tag{2}$$

Dividing both sides of Eq. (2) by the total protein mass we obtain an equation for the instantaneous growth rate $\lambda(t)$:

$$\lambda(t) = \frac{1}{M(t)}\frac{{\rm d}M(t)}{{\rm d}t} = \sigma(t)\phi_{\rm R}(t) \ . \tag{3}$$

The translational efficiency σ is an average translation rate, and does not distinguish between active and inactive ribosomes. If we consider that at any moment only a fraction $f_{\rm act} = M_{\rm Rb}^{\rm act}/M_{\rm Rb}$ is translating at a rate $k_{\rm el}$, we may write:

$$\frac{{\rm d}M}{{\rm d}t} = k_{\rm el} M_{\rm R}^{\rm act} = k_{\rm el} f_{\rm act} M_{\rm R} \equiv \sigma M_{\rm R} \ . \tag{4}$$

Therefore, σ can be decomposed as the product of the elongation rate of the active ribosomes and the active ribosome fraction, $\sigma(t) = k_{\rm el}(t) \cdot f_{\rm act}(t)$. A recent study [6] highlighted the contribution of the translation rate $k_{\rm el}$ and the fraction of active ribosomes $f_{\rm act}$ to σ. As the nutrient source is decreased from rich to poor media, the steady state elongation rate $k_{\rm el}$ drops from ~ 17 aa/s in fast growth to about 8 aa/s in slow growth or stationary state. Given the elongation rates $k_{\rm el}$, the fraction of active ribosomes $f_{\rm act}$ were computed from the known ribosome abundances and the protein flux. While at fast growth about 90% of the ribosomes are active, this number decreases down to 20% for very slow growth [6].

In the upshift experiments performed in this work we notice that growth rate increases up to eightfold in the few minutes after upshift. As can be seen in Main Text Figure 2b, where the culture was shifted from aspartate ($\lambda_{\rm i} = 0.06$/h) to rich media, growth rate increases to $\lambda_0 \approx 0.50$ in a few minutes. Since

protein abundances cannot change significantly in this short period, the increase of growth rate must orginate in an increase of the the translational efficiency $\sigma(t)$ by a factor $\lambda_0/\lambda_\mathrm{i} \approx 8$. Such an 8-fold increase of the translational efficiency requires both an increase in elongation rate k and an activation of previously inactivated ribosomes f_act.

1.2 Protein allocation

In order to solve how the increase of the translational efficiency impacts the growth transition kinetics, we describe the protein synthesis flux. The total synthesis flux $\mathrm{d}M/\mathrm{d}t$ is allocated to different protein sectors j, under the condition that the protein synthesis fractions χ_j sum up to unity,

$$\sum_j \chi_j(t) = 1 \ . \tag{5}$$

The values of $\chi_j(t)$ are set by several factors, such as the abundance of mRNA of the species j and/or the translation efficiency of the specific transcript. Using the total protein synthesis, Eq. (2), the synthesis of a protein section j is given by

$$\frac{\mathrm{d}M_j(t)}{\mathrm{d}t} = \chi_j(t)\frac{\mathrm{d}M(t)}{\mathrm{d}t} = \chi_j(t)\sigma(t)M_\mathrm{R}(t) \ , \tag{6}$$

From this equation, we can compute the time derivative of the protein fraction ϕ_j:

$$\frac{\mathrm{d}\phi_j(t)}{\mathrm{d}t} = \frac{\mathrm{d}}{\mathrm{d}t}\left(\frac{M_j(t)}{M(t)}\right) = \frac{1}{M(t)}\frac{\mathrm{d}M_j(t)}{\mathrm{d}t} - \frac{M_j(t)}{M(t)}\frac{\mathrm{d}M(t)}{\mathrm{d}t} \tag{7}$$

Using equations (2) and (6) we obtain a logistic equation for the proteome fractions:

$$\frac{\mathrm{d}\phi_j(t)}{\mathrm{d}t} = \sigma(t)\phi_\mathrm{R}(t)\left(\chi_j(t) - \phi_j(t)\right) = \lambda(t)\left(\chi_j(t) - \phi_j(t)\right) \ . \tag{8}$$

The above equations describe the dynamics of growth, Eq. (3) and of the protein fractions, Eq. (8). They are determined by the translation rate $\sigma(t)$ and on the synthesis fractions $\chi_j(t)$. The knowledge of these functions allows solving the dynamic system. In particular, specializing this equation to the R-proteins, we see that ϕ_R depends on $\sigma(t)$ and $\chi_\mathrm{R}(t)$ only:

$$\frac{\mathrm{d}\phi_\mathrm{R}(t)}{\mathrm{d}t} = \sigma(t)\phi_\mathrm{R}(t)\left(\chi_\mathrm{R}(t) - \phi_\mathrm{R}(t)\right) \ . \tag{9}$$

If σ and χ_j are constant in time, Eq. (8) directly gives the steady state values of the mass fractions, $\phi_j^* = \chi_j$, and the growth rate, $\lambda^* = \sigma\chi_\mathrm{R}$.

Let us note that, even for instantaneous variations of the regulatory functions $\chi_j(t)$, the mass fractions $\phi_j(t)$ do not adjust immediately. Instead, the timescale is set by the growth rate $\lambda(t)$, as can be checked rewriting Eq. (8) in terms of $\Delta_j(t) = \chi_j(t) - \phi_j(t)$:

$$\frac{\mathrm{d}}{\mathrm{d}t}\Delta_j(t) = -\lambda(t)\Delta_j(t) + \frac{\mathrm{d}}{\mathrm{d}t}\chi_j(t) \ . \tag{10}$$

which is a first-order differential equation for $\Delta_j(t)$, driven by an external "force" $\mathrm{d}\chi_j/\mathrm{d}t$. After a change in χ_j, Δ_j relaxes toward zero with a rate $\dot{\Delta}_j/\Delta_j = -\lambda(t)$ for all protein sectors j.

1.3 Solution of the instantaneous model

As described in the main text, the experimental findings of Schleif and Dennis & Bremer [1, 2, 3] showed that the the total protein synthesis rate and the ribosome synthesis rate increase within a few minutes to their final values after an upshift to rich medium. These fast kinetics can be approximated by an instantaneous increase of the of the translational efficiency σ and the R-protein synthesis fraction χ_{R}.

$$\sigma(t) = \begin{cases} \sigma_{\mathrm{i}} \ , & t < 0 \ , \\ \sigma_{\mathrm{f}} \ , & t \geq 0 \ , \end{cases} \qquad \chi_{\mathrm{R}}(t) = \begin{cases} \chi_{\mathrm{R}}^{\mathrm{i}} \ , & t < 0 \ , \\ \chi_{\mathrm{R}}^{\mathrm{f}} \ , & t \geq 0 \ . \end{cases} \tag{11}$$

where the sub and superscripts indicate the initial and final states. Under these assumptions, Eq. (9) can be readily solved, obtaining $\phi_{\mathrm{R}}(t)$ as:

$$\phi_{\mathrm{R}}(t) = \begin{cases} \chi_{\mathrm{R}}^{\mathrm{i}} \ , & t < 0 \ , \\ \dfrac{\chi_{\mathrm{R}}^{\mathrm{f}}}{1 - \left(1 - \chi_{\mathrm{R}}^{\mathrm{f}}/\phi_{\mathrm{R}}^{\mathrm{i}}\right) \exp\left(-\lambda_{\mathrm{f}} t\right)} \ , & t \geq 0 \ . \end{cases} \tag{12}$$

The growth rate $\lambda(t)$ is then obtained by combining the equation above and the relationship $\lambda(t) = \sigma(t)\phi_{\mathrm{R}}(t)$, Eq. (3), yielding Main Text Equation (6). The mass increase $M(t)/M(0)$ can then be computed by integrating $\lambda(t)$ over time. However, another derivation of the same equations might allow for a better understanding of the growth kinetics. Specializing Eq. (6) for the R-proteins, we see that the accumulation of R-proteins is exponential, with pre-shift rate $\lambda_{\mathrm{i}} = \sigma_{\mathrm{i}}\chi_{\mathrm{R}}^{\mathrm{i}}$ and post-shift rate $\lambda_{\mathrm{f}} = \sigma_{\mathrm{f}}\chi_{\mathrm{R}}^{\mathrm{f}}$:

$$M_{\mathrm{R}}(t) = \begin{cases} M_{\mathrm{R}}(0)e^{\lambda_{\mathrm{i}} t} \ , & t \leq 0 \ , \\ M_{\mathrm{R}}(0)e^{\lambda_{\mathrm{f}} t} \ , & t \geq 0 \ . \end{cases} \tag{13}$$

Here $M_{\mathrm{R}}(0) = \chi_{\mathrm{R}}^{\mathrm{i}} M(0)$ is the mass of R-proteins at time $t = 0$. We can then plug expression into Eq. (2), which can be integrated to obtain the kinetics for the total protein mass, Main Text Eq. (7):

$$\frac{M(t)}{M(0)} = \begin{cases} e^{\lambda_{\mathrm{i}} t} \ , & t \leq 0 \ , \\ 1 + \frac{\lambda_0}{\lambda_{\mathrm{f}}}\left(e^{\lambda_{\mathrm{f}} t} - 1\right) \ , & t \geq 0 \ , \end{cases} \tag{14}$$

with $\lambda_0 = \lambda_{\mathrm{f}}\chi_{\mathrm{R}}^{\mathrm{i}}/\chi_{\mathrm{R}}^{\mathrm{f}} = \sigma_{\mathrm{f}}\chi_{\mathrm{R}}^{\mathrm{i}}$. Given $M(t)$ and $M_{\mathrm{R}}(t)$, we can now compute both $\phi_{\mathrm{R}}(t)$ and $\lambda(t)$ from their definitions; for the growth rate, we again obtain Main Text Eq. (6) by taking the logarithmic derivative of $M(t)$. Note that both the protein fractions and the growth rate kinetics take a particularly simple form when expressed as a function of $W(t) = M(t)/M(0)$:

$$\phi_{\mathrm{R}}(t) = \chi_{\mathrm{R}}^{\mathrm{i}} + (\chi_{\mathrm{R}}^{\mathrm{f}} - \chi_{\mathrm{R}}^{\mathrm{i}})\left(1 - \frac{1}{W(t)}\right) \quad (t \geq 0) \tag{15}$$

$$\lambda(t) = \lambda_0 + (\lambda^{\mathrm{f}} - \lambda_0)\left(1 - \frac{1}{W(t)}\right) \quad (t \geq 0) \tag{16}$$

This form makes evident how the growth kinetics after the shift are simply set by biomass accumulation. For instance, at one doubling after the shift ($W = 2$) the protein fractions are half-way between the pre-shift and the post-shift values, $\phi_{\mathrm{R}}(W = 2) = (\phi_{\mathrm{R}}^{\mathrm{i}} + \phi_{\mathrm{R}}^{\mathrm{f}})/2$. Eq. (15) also holds for a generic protein fraction $\phi_j(t)$, as long as its synthesis fraction $\chi_j(t)$ also changes instantaneously ($\chi_j^{\mathrm{i}} \to \chi_j^{\mathrm{f}}$) at the shift.

The upshift kinetics can be summarized as follows:

- The first phase is a rapid increase in the instantaneous growth rate from $\lambda_\mathrm{i} = \sigma_\mathrm{i}\phi_\mathrm{R}^\mathrm{i}$ to a larger value $\lambda_0 = \sigma_\mathrm{f}\phi_\mathrm{R}^\mathrm{i}$. The jump in growth rate stems from an increase in translational efficiency:

$$\lambda_0 - \lambda_\mathrm{i} = \big(\sigma_\mathrm{f} - \sigma_\mathrm{i}\big)\phi_\mathrm{R}^\mathrm{i} \ . \tag{17}$$

- The second phase is a much slower increase in the growth rate from $\lambda_0 = \sigma_\mathrm{f}\phi_\mathrm{R}^\mathrm{i}$ to the final value $\lambda_\mathrm{f} = \sigma_\mathrm{f}\phi_\mathrm{R}^\mathrm{f}$. This time, the further increase in growth rate is due to the increase in ribosomes, with no variation in the translational efficiency:

$$\lambda_\mathrm{f} - \lambda_0 = \sigma_\mathrm{f}\big(\phi_\mathrm{R}^\mathrm{f} - \phi_\mathrm{R}^\mathrm{i}\big) \ . \tag{18}$$

Lag time

The quantity λ_0, is related to the lag time τ_lag. The relative mass increase $W(t) = M(t)/M(0)$, Main Text Eq. (7) or Eq. (14), can be written as:

$$W(t \to \infty) \sim \frac{\lambda_0}{\lambda_\mathrm{f}} \exp(\lambda_\mathrm{f} t) \ . \tag{19}$$

The lag time is defined as $W(t \to \infty) = \exp(\lambda_\mathrm{f}(t - \tau_\mathrm{lag}))$, therefore, we can solve for the lag time and obtain

$$\tau_\mathrm{lag} = \frac{1}{\lambda_\mathrm{f}} \log\left(\frac{\lambda_\mathrm{f}}{\lambda_0}\right) = \frac{1}{\lambda_\mathrm{f}} \log\left(\frac{\chi_\mathrm{R}^\mathrm{f}}{\chi_\mathrm{R}^\mathrm{i}}\right) \ . \tag{20}$$

or vice-versa, λ_0 in terms of the lag time τ_lag:

$$\lambda_0 = \lambda_\mathrm{f} e^{-\lambda_\mathrm{f}\tau_\mathrm{lag}} \ . \tag{21}$$

Supplementary Note 2: Upshift fitness landscape

The aim of this Note is to derive the fitness of strains with different ϕ_{R0}, exposed to different environments. We will restrict ourselves to the simple situation in which the cell culture undergoes a nutritional upshift at time $t = 0$, that lasts a fixed amount of time T. We study the fitness, or relative mass increase $W(T) = M(T)/M(0)$ during this feast period. This models a famine and feast environment in which the cells are supplied with rich nutrients (feast) for a time interval T, followed by long periods of no-growth (famine). The strain which produces more mass in the time window has the selective advantage over the others.

Adaptation kinetics is set by λ_0, the growth rate "immediately" after the shift, and the asymptotic growth rate λ_{f}. To see the importance of λ_0, let us consider two identical cell populations, whose growth kinetics are both described by Eq. (14), undergoing an upshift at time $t = 0$. In the simple case in which the initial populations are the same ($M^{(1)}(0) = M^{(2)}(0)$) and the final growth rates are the same ($\lambda_{\mathrm{f}}^{(1)} = \lambda_{\mathrm{f}}^{(2)} \equiv \lambda_{\mathrm{f}}$), one can compute the ratio of the bacterial (protein) masses using Eq. (14) as:

$$\frac{M^{(1)}(t)}{M^{(2)}(t)} = \frac{W^{(1)}(t)}{W^{(2)}(t)} = \frac{1 + (\lambda_0^{(1)}/\lambda_{\mathrm{f}})(e^{\lambda_{\mathrm{f}} t} - 1)}{1 + (\lambda_0^{(2)}/\lambda_{\mathrm{f}})(e^{\lambda_{\mathrm{f}} t} - 1)} \tag{22}$$

From this equation we can see that the mass ratio asymptotically reaches the value $\lambda_0^{(1)}/\lambda_0^{(2)}$ as $T \gg \lambda_{\mathrm{f}}$. This simple example shows that a large value of λ_0, and thus of the ribosomal overcapacity ϕ_{R0}, may provide a large increase in the mass of the cell population in dynamic environments. However, if the final growth rates $\lambda_{\mathrm{f}}^{(1)}$ and $\lambda_{\mathrm{f}}^{(2)}$ are not equal, the fastest growing strain will always dominate after a long time from the shift.

2.1 Cost of the R-sector overcapacity

To model the reduction of growth rate due to an R-protein overcapacity, we use the empirical relation of growth rate established for different levels of useless protein overexpression [4], which states that growth rate linearly decreases with the amount of useless proteins. Given that the wild type strain, with overcapacity $\phi_{\mathrm{R0}}^{\mathrm{WT}}$, has a growth rate λ^{WT} (we suppress the asterisk in order not to overload the notation), the growth rate of a strain with a different overcapacity ϕ_{R0} is given by:

$$\lambda^*(\phi_{\mathrm{R0}}) = \lambda^{\mathrm{WT}} \frac{\phi_{\mathrm{R}}^{\max} - \phi_{\mathrm{R0}}}{\phi_{\mathrm{R}}^{\max} - \phi_{\mathrm{R0}}^{\mathrm{WT}}} \ , \tag{23}$$

where $\phi_{\mathrm{R}}^{\max}$ is empirically determined to be close to 48% [4]. This expression can be rearranged in the following equivalent form, more useful for an analysis of the fitness landscape:

$$\frac{\lambda^*(\phi_{\mathrm{R0}})}{\phi_{\mathrm{R}}^{\max} - \phi_{\mathrm{R0}}} = \frac{\lambda^{\mathrm{WT}}}{\phi_{\mathrm{R}}^{\max} - \phi_{\mathrm{R0}}^{\mathrm{WT}}} \ , \tag{24}$$

which means that the r.h.s. of the equation does not depend on the offset ϕ_{R0}. We can divide the above equation by ν_{R}, thus allowing us to define a dimensionless constant z^*,

$$z^* = \frac{\lambda^*}{\nu_{\mathrm{R}}(\phi_{\mathrm{R}}^{\max} - \phi_{\mathrm{R0}})} = \frac{\lambda^*}{\lambda_{\max}} \ . \tag{25}$$

where $\lambda_{\max}$ is the growth rate in rich media (see Main Text Eq. (10)). Note that z^* is independent of ϕ_{R0}. The quantity z^* reflects the quality of the nutrients, with $z^* = 1$ corresponding to rich media,

and lower values to poorer media. In the case of upshifts, the two steady states are described by two nutrient qualities $z_i < z_f$. We can then express the growth rate λ^* for different nutrient levels and different offsets as:

$$\lambda^* = z^* v_\mathrm{R} \left(\phi_\mathrm{R}^\mathrm{max} - \phi_\mathrm{R0}\right) = z^* \left(\lambda_\mathrm{R}^\mathrm{max} - \lambda_\mathrm{R0}\right) \tag{26}$$

where $\lambda_\mathrm{R}^\mathrm{max} = \nu_\mathrm{R} \phi_\mathrm{R}^\mathrm{max}$ is close to 2.9/h for our strain. This value can be computed from the known offset and maximum growth rates of our strain as $\lambda_\mathrm{R}^\mathrm{max} = \lambda_\mathrm{max}^* + \lambda_\mathrm{R0}$, where $\lambda_\mathrm{R0} \approx 0.44$/h (see Fig. S1a) and $\lambda_\mathrm{max}^* \approx 2.45$/h (growth rate in rich media). (The value of $\phi_\mathrm{R}^\mathrm{max} = 48\%$ can be then obtained as $\lambda_\mathrm{R}^\mathrm{max}/\nu_\mathrm{R}$, with $\nu_\mathrm{R} \approx 6$/h, see again Fig. S1).

The growth rate λ^WT of the wild type strain can be thus expressed in terms of z^* and the wild type overcapacity through Eq. (26); the same relation can be inverted, if needed, to express z^* in terms of λ^WT and $\phi_\mathrm{R0}^\mathrm{WT}$. In the following we will use the more convenient z^* to study the fitness landscape.

2.2 Relation to the Scott et al. model

Equation (26) can be seen as a specialization of the protein allocation model introduced by Scott and coworkers in Ref. [4] to the case of different nutrient, without considering the case of translation inhibition. In Ref. [4], the proteome is partitioned into three sectors P, R and Q, with mass fractions ϕ_P, ϕ_R an ϕ_Q, respectively. As in this work, ϕ_R gathers ribosomal proteins and other proteins which are coexpressed with ribosomes ("affiliated"). The P- and R-sectors depend on the steady state growth rate λ^* as:

$$\phi_\mathrm{P} = \lambda^*/\kappa_\mathrm{n} \tag{27}$$

$$\phi_\mathrm{R} = \phi_\mathrm{R0} + \lambda^*/\kappa_\mathrm{t} \tag{28}$$

Here, κ_n is the "nutritional capacity" of the cell, and is set by the nutrient quality of the medium the cells grow in. The "translational capacity" κ_t of the cell is independent on the nutrients, but is reduced as translation-limiting antibiotics are added to the medium. Eq. (28) is analogous to Main Text Eq. (1) if we identify $\kappa_\mathrm{t} = \nu_\mathrm{R}$. The third sector, with mass fraction ϕ_Q, is taken to be constant. The sum of all proteome sectors has to sum up to one, $\phi_\mathrm{P} + \phi_\mathrm{R} + \phi_\mathrm{Q} = 1$, see (1); this constraint allows to compute growth rate as a function of the parameters κ_n and κ_t as follows:

$$\lambda^* = \left(1 - \phi_\mathrm{Q} - \phi_\mathrm{R0}\right) \frac{\kappa_\mathrm{n}\kappa_\mathrm{t}}{\kappa_\mathrm{n} + \kappa_\mathrm{t}} \tag{29}$$

There is a 1:1 relationship between the quantities appearing in Eq. (26) and the ones considered in Scott *et al.* A direct comparison reveals:

$$\phi_\mathrm{R0}^\mathrm{max} = 1 - \phi_\mathrm{Q} \qquad , \qquad z^* = \frac{\kappa_\mathrm{n}}{\kappa_\mathrm{n} + \kappa_\mathrm{t}} \ . \tag{30}$$

These relationships form a 1:1 mapping between the two notations. However, in the following, it will be more convenient to use the notation of Eq. (26).

2.3 Analysis of the fitness landscape

The upshifts in rich media are fully specified described by the kinetics derived in Supp. Note 1. The absolute fitness is defined as the relative mass increase, $W(T) = M(T)/M(0)$ (Main Text Eq. (7) and Eq. (14) in Supplementary Note 1) after a feast period T. By plugging the R-sector growth law into $W(t)$, we obtain:

$$W(T) = 1 + \frac{\lambda_\mathrm{R0} + \lambda_\mathrm{i}}{\lambda_\mathrm{R0} + \lambda_\mathrm{f}} \left(e^{\lambda_\mathrm{f} T} - 1\right) \quad \text{for } T \geq 0 \ , \tag{31}$$

where the initial and final growth rates are functions of the nutrient qualities z and the overcapacities/offsets as in Eq. (26). An alternate form for the same quantity can be obtained in terms of the lag time τ_{lag}, defined in Eq. (20):

$$W = 1 + \left(e^{-\lambda_{\text{f}}\tau_{\text{lag}}}\right)\left(e^{\lambda_{\text{f}}T} - 1\right) \ . \tag{32}$$

In principle, W is a function of three environmental variables T, z_{i} and z_{f}, and three strain variables λ_{R0} (or ϕ_{R0}), $\phi_{\text{R}}^{\text{max}}$ and ν_{R}. However, dynamics is specified by less than six variables. We define the non-dimensional parameters

$$x = \frac{\phi_{\text{R0}}}{\phi_{\text{R}}^{\text{max}}} = \frac{\lambda_{\text{R}}}{\lambda_{\text{R}}^{\text{max}}} \ , \quad \tau = \lambda_{\text{R}}^{\text{max}} T \ . \tag{33}$$

These quantities allow us to rewrite Eqs. (26) and (31) with only four variables, instead of six. The fitness W is therefore only a function of the rescaled R–sector offset, $x = \lambda_{\text{R}}/\lambda_{\text{R}}^{\text{max}}$, the rescaled feast time $\tau = \lambda_{\text{R}}^{\text{max}} T$ and the initial and final nutrient source qualities z_{i} and z_{f}. Equation (31) reduces to a very simple form:

$$W(x|\tau, z_{\text{i}}, z_{\text{f}}) = 1 + \frac{x + z_{\text{i}}(1-x)}{x + z_{\text{f}}(1-x)}\Big(e^{(1-x)z_{\text{f}}\tau} - 1\Big) \ . \tag{34}$$

The optimal value of the overcapacity $x^\star(\tau, z_{\text{i}}, z_{\text{f}})$ for a given environment – described by τ, z_{i} and z_{f} – is obtained by setting to zero the partial derivative of W with respect to x, yielding the following equation:

$$\big(z_{\text{i}} + (1-z_{\text{i}})x^\star\big)\big(z_{\text{f}} + (1-z_{\text{f}})x^\star\big) = \big(z_{\text{f}} - z_{\text{i}}\big)\frac{1 - e^{-z_{\text{f}}\tau(1-x^\star)}}{z_{\text{f}}\tau} \tag{35}$$

This equation cannot be solved in closed form, however, it is possible to obtain simple expressions in a few limiting cases, such as in the limit of either small or large τ.

2.3.1 Small time limit, upshift to rich media

In this case consider the limit $\tau \to 0$, and we will only consider upshifts to high quality nutrients, that is, $\nu_{\text{P}}^{\text{f}} \gg \nu_{\text{R}}$, or $z_{\text{f}} = 1$. For small τ, one can approximate $e^{(1-x)\tau} - 1 \sim (1-x)\tau$, thus obtaining:

$$W \sim 1 + (x + z_{\text{i}}(1-x))(1-x)\tau \ , \tag{36}$$

$$\frac{\partial W}{\partial x} \sim 2\tau(1-z_{\text{i}})\Big(\frac{1-2z_{\text{i}}}{2(1-z_{\text{i}})} - x\Big) \ , \tag{37}$$

$$x^\star(\tau \to 0, z_{\text{i}}) \sim \frac{1-2z_{\text{i}}}{2(1-z_{\text{i}})} \ . \tag{38}$$

or

$$\lambda_{\text{R0}}^\star \sim \frac{1-2z_{\text{i}}}{2(1-z_{\text{i}})}\lambda_{\text{R}}^{\text{max}} \ . \tag{39}$$

This condition favors the largest possible offset for very low nutritional quality ($z_{\text{i}} = 0$, corresponding to $x^\star = 1/2$), while the optimal offset goes to zero when $z_{\text{i}} = 1/2$. Given that the value of the offset maximizing the fitness is a monotonously decreasing function of the feast time T (see e.g. Fig. 4a in the Main Text), the strains with $x > 1/2$, or $\lambda_{\text{R0}} > \lambda_{\text{R}}^{\text{max}}$, are suboptimal in any environmental condition.

2.3.2 Large time limit

In the opposite limit, i.e. $\tau \gg 1$, one can write $e^{(1-x)\tau} - 1 \sim e^{(1-x)\tau}$. We will discuss the limits of validity of this approximation below. By taking the derivative $\partial W/\partial x$ and setting it to zero, we obtain a quadratic equation for $x^\star$:

$$a(x^\star)^2 + bx^\star - c = 0 \ , \tag{40}$$

with

$$a = (1 - z_\mathrm{i})(1 - z_\mathrm{f}) \ , \quad b = z_\mathrm{i} + z_\mathrm{f} - 2z_\mathrm{i}z_\mathrm{f} \ , \quad c = \frac{z_\mathrm{f} - z_\mathrm{i}}{z_\mathrm{f}\tau} - z_\mathrm{i}z_\mathrm{f} \ . \tag{41}$$

We are looking for positive solutions to the equation, which is only possible for $c > 0$, or

$$T < T_c = \frac{z_\mathrm{f} - z_\mathrm{i}}{z_\mathrm{i}z_\mathrm{f}^2}\lambda_\mathrm{R}^\mathrm{max} \ . \tag{42}$$

Therefore, the optimal offset is larger than zero if $T < T_c$, and equal to zero if $T \geq T_c$. This relation shows that, for any given value of $z_\mathrm{i} > 0$, the strains with no offset ($x = 0$) become optimal in a finite time T_c. At steady state, we know that the strain with no offset has the largest growth rate (by directly inspecting from Eq. (26)). We thus recover the steady state solution either if we take T to be larger than T_c, or if we set $z_\mathrm{i} = z^\mathrm{f}$, i.e. there is no upshift at all. However, the critical time T_c diverges when $z_\mathrm{i} = 0$, i.e. for upshifts from vanishing initial growth rates. This implies that the strain with no offset is never optimal for any finite feast time T. The reason is that such strain would have no ribosomes at all in the pre-shift condition, and therefore its growth rate will be zero at all times!

In the large time limit, $T \to T_c$, one has $c = z_\mathrm{i}z_\mathrm{f}(T_c/T - 1) \to 0$. Instead, if $z_\mathrm{i} \to 0$, then $a \to 0$. In both cases, the solution of the equation is approximately given by $x^\star = c/b$, plus terms of order $a \cdot c/b^2$. In terms of z_i, z_f and τ:

$$x^\star = \frac{c}{b} = \frac{1 - z_\mathrm{i}/z_\mathrm{f}}{z_\mathrm{i} + z_\mathrm{f} - 2z_\mathrm{i}z_\mathrm{f}}\frac{1}{\tau} - \frac{z_\mathrm{i}z_\mathrm{f}}{z_\mathrm{i} + z_\mathrm{f} - 2z_\mathrm{i}z_\mathrm{f}} \ . \tag{43}$$

This expression is not very informative. We will describe the two limits $z_\mathrm{i} \to 0$ and $z_\mathrm{f} \to 1$, as well as the combination of the two. In this last case, we set $z_\mathrm{i} = 0$ and $z_\mathrm{f} = 1$ in Eq. (43), obtaining Equation (11) from the Main Text:

$$x^\star = \frac{1}{\tau} \quad \Rightarrow \quad \lambda_\mathrm{R0}^\star = \frac{1}{T} \quad (z_\mathrm{i} \to 0 \ , \ z_\mathrm{f} \to 1) \ . \tag{44}$$

If the initial growth rate is not zero, the optimal offset is linearly reduced by $1/T_c$, therefore going to zero for large enough times:

$$\lambda_\mathrm{R0}^\star = \frac{1}{T} - \frac{1}{T_c} \ , \quad \frac{1}{T_c} = \frac{z_\mathrm{i}}{1 - z_\mathrm{i}}\lambda_\mathrm{R}^\mathrm{max} \quad (z_\mathrm{f} \to 1) \ . \tag{45}$$

If instead the initial growth rate is zero, but the final nutrient quality is reduced, the optimal value of the offset is increased by a factor $1/z_\mathrm{f}$, corresponding to the decrease in the post-shift growth rate:

$$\lambda_\mathrm{R0}^\star = \frac{1}{z_\mathrm{f}} \times \frac{1}{T} \quad (z_\mathrm{i} \to 0) \ . \tag{46}$$

The expressions above have been computed by assuming $\exp(z_\mathrm{f}\tau(1-x)) = \exp(\lambda_\mathrm{f}T) \gg 1$. Corrections to these expressions are be exponentially small, as can be seen in the case $z_\mathrm{i} = 0$, $z_\mathrm{f} = 1$. In this case, we can rewrite the equation $\partial W/\partial x = 0$ as:

$$(1 - \tau x)e^{1-\tau x} = e^{1-\tau} \ , \tag{47}$$

which is solved in terms of Lambert's W function (which we denote as W_L) as:

$$1 - \tau x = W_L \left(e^{1-\tau}\right) \ . \tag{48}$$

Lambert's W function admits a power series expansion around 0 as $W_L(z) \sim z - z^2 + \ldots$, with a convergence radius of $1/e$. Therefore, one obtains:

$$x = \frac{1}{\tau}\left(1 - W_L\left(e^{1-\tau}\right)\right) \sim \frac{1}{\tau}\left(1 - e^{1-\tau} + e^{2(1-\tau)} + \ldots\right) \tag{49}$$

The series converges quickly for $\tau > 2$, or $T > 2/\lambda_{\mathrm{R}}^{\mathrm{max}} \sim 0.6$ h.

Supplementary Figures

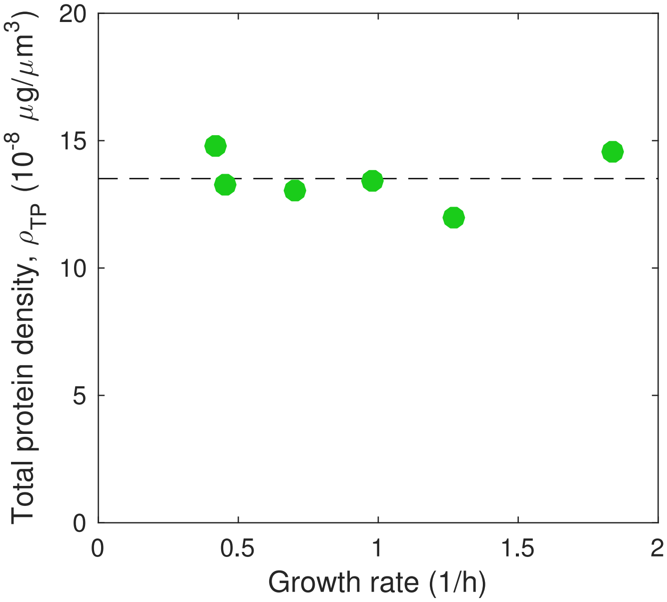

Supplementary Figure 1: Protein density for exponentially growing cells. Density of total proteins ρ_{TP} as a function of growth rate, for different nutrient sources; the dashed line indicates the average value $\rho_{\mathrm{TP}} = 13.51 \times 10^{-8} \mu\mathrm{g}/\mu\mathrm{m}^3$. The total density ρ_{TP} has been computed using data from Basan et al. (2015) [9] as $\rho_{\mathrm{TP}} = M_{\mathrm{TP}}/(N \cdot V)$, where M_{TP} is the total protein mass per OD, N is the number of cells per OD, and V is the average cell volume. For a given protein i with protein mass fraction ϕ_i, the correspondent density ρ_i (protein mass per volume) is simply given by $\rho_i = \phi_i \rho_{\mathrm{TP}}$; the concentration (copy number per volume) is then obtained by dividing ρ_i by the protein mass.

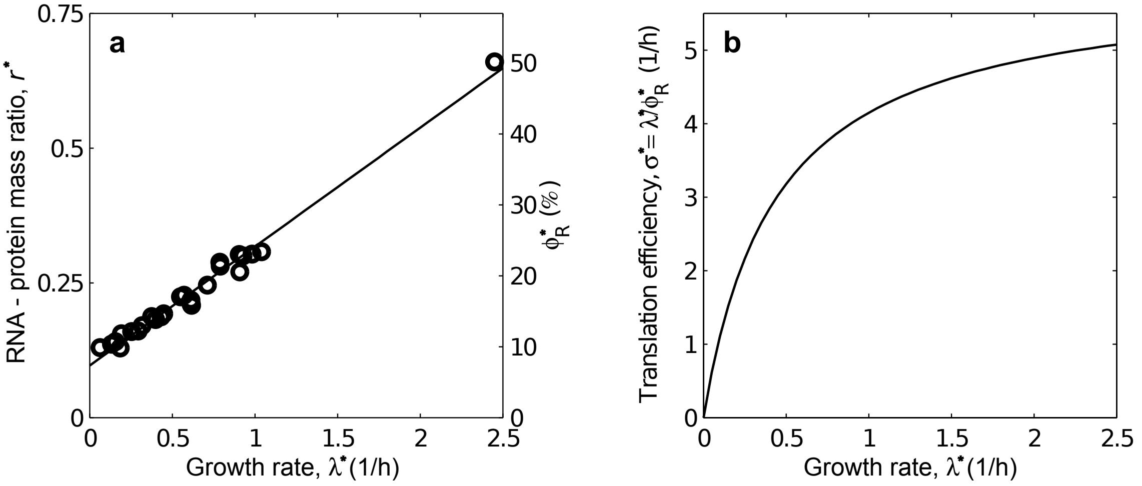

Supplementary Figure 2: RNA to protein ratio and translational efficiency. (**a**) Ratio of total RNA mass to total protein mass, r^*, as a function of the steady state growth rate, λ^*. Each point corresponds to a different nutrient source; The right-most point corresponds to rich media (LB + 0.2% glucose, $\lambda^* = 2.45/h$). The black line is a linear fit $r^* = a + b\lambda^*$ to the data, with $a = 0.0967$ and $b = 0.2206$ h. The right axes shows the correspondent R-sector protein fraction, using $\phi^*_{\mathrm{R}} = \rho r^*$ with $\rho = 0.76$ (from Scott et al (2010) [4]). (**b**) Steady state translation efficiency $\sigma^* = \lambda^*/\phi^*_{\mathrm{R}}$, computed from the mRNA-protein mass ratio with the same conversion factor as before. The line is a Michaelis-Menten function of the growth rate, with equation $\sigma^* = \nu_{\mathrm{R}}\lambda^*/(\lambda^* + \lambda_{\mathrm{R0}})$, computed using the same parameters as the line in panel (a), i.e. $\nu_{\mathrm{R}} = 1/(\rho b) \approx 6.0$ h^{-1} and $\lambda_{\mathrm{R0}} = a/b \approx 0.43$ h^{-1}.

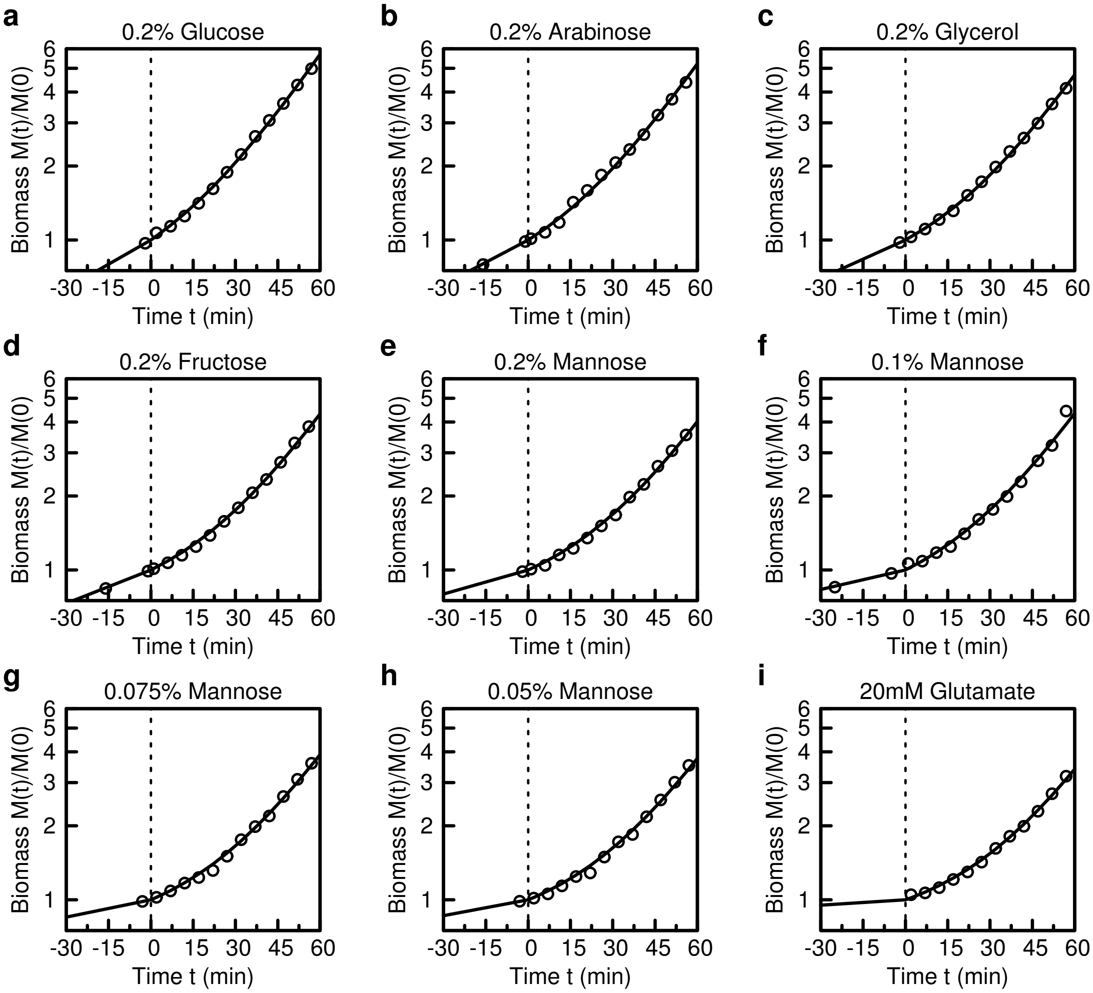

Supplementary Figure 3: Mass accumulation in upshifts to rich medium. (**a-i**) Cells grown exponentially on N-C- minimal medium supplemented with 20mM NH_4 and the carbon substrate indicated above panel are subjected to an upshift to rich medium (LB + 0.2% glucose) at $t = 0$ (symbols). The solid line represents, for $t > 0$, Eq. (7) in main text, using $\lambda_f = 2.45$/h and the fit results in Supplementary Table 2 for λ_0. The standard deviation of the fit residuals is between 0.006 to 0.013 for the various upshifts, yielding error bars roughly the size of the markers.

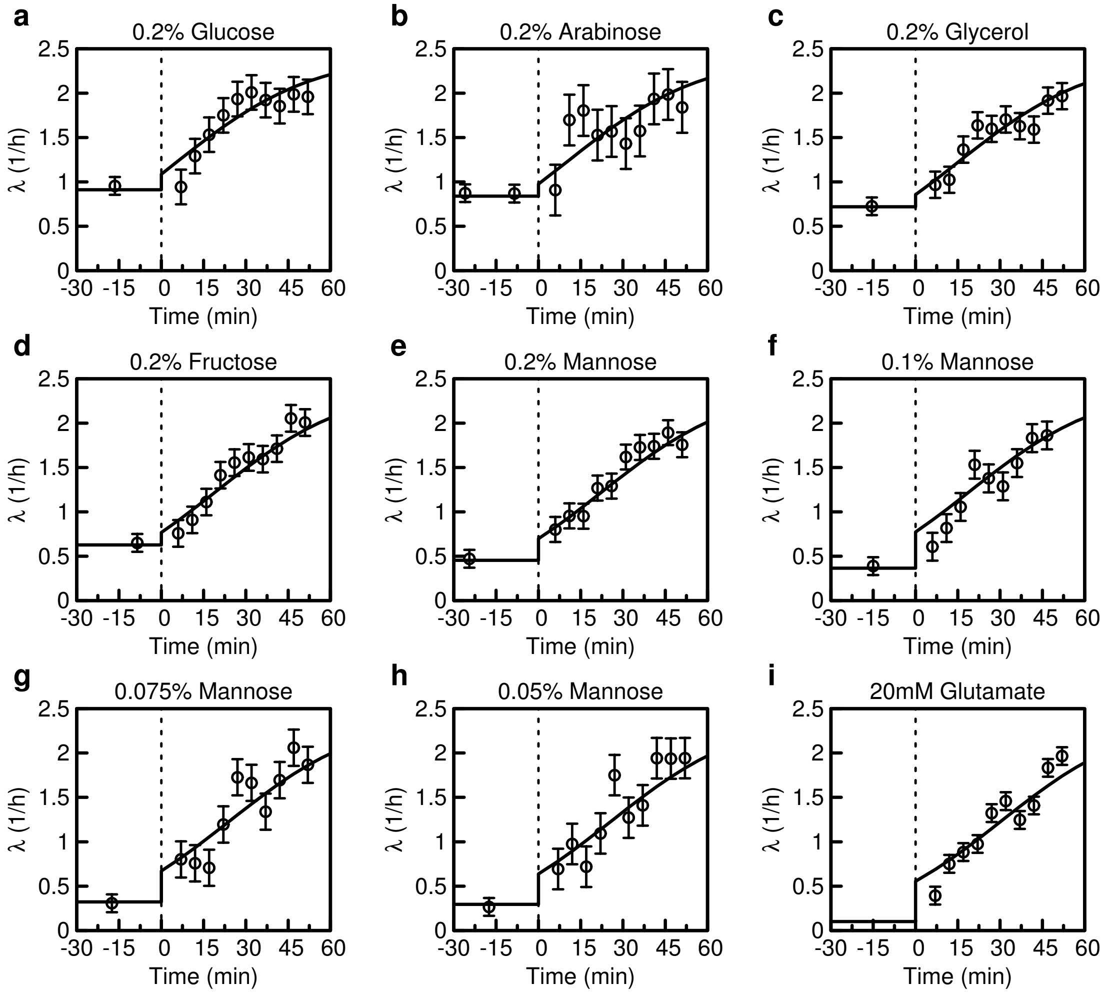

Supplementary Figure 4: Instantaneous growth rate in upshifts to rich medium. (**a-i**) Cells grown exponentially on N-C- minimal medium supplemented with 20mM NH_4 and the carbon substrate indicated above panel are subjected to an upshift to rich medium (LB + 0.2% glucose) at $t = 0$ (symbols). The points are computed from the experimental OD measurements, Fig. 3, by taking the best fitting slope between triplets of adjacent points, yielding $\lambda_k = (\log M_{k+1} - \log M_{k-1})/2\Delta t$. The error bar is propagated from the average residuals of $\log M_k$, σ_r, as $\sigma_\lambda = \sqrt{2}\sigma_r/2\Delta t$. The solid line represents Eq. (6) from the Main Text, using the the final growth rate $\lambda_\text{f} = 2.45$/h and the best fitting λ_0; fit results, summarized in Supplementary Table 2, are plotted in Fig. 2c of the main text.

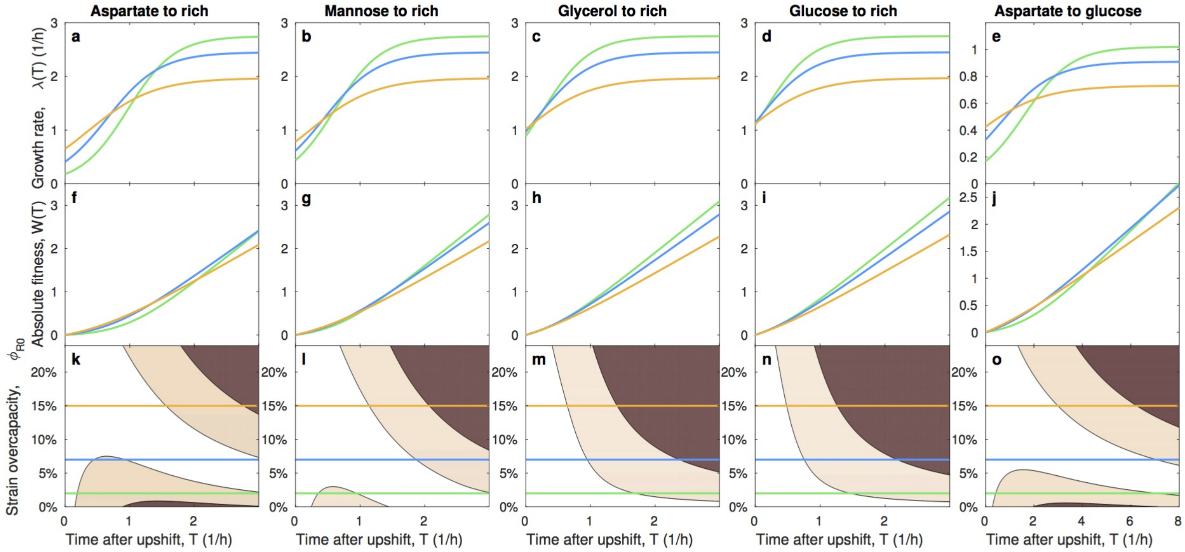

Supplementary Figure 5: Fitness landscape for several nutrient shifts. (**a-e**) Growth rate after the shift (at time $T = 0$), for three strains with different overcapacities ($\phi_{\mathrm{R0}} = 2\%$, 7%, 15%, as in Fig. 3ab), for five different upshifts: four shifts from poor media (aspartate, mannose, glycerol ad glucose, with pre-shift growth rates λ_{i} =0.06/h, 0.3/h, 0.73/h, 0.91/h, respectively, as in Main Text Fig. 4ab) to rich media, and a shift from slow growth ($\lambda_{\mathrm{i}} = 0.06/h$) to an intermediate growth rate (the one yielded by glucose, $\lambda_{\mathrm{f}} = 0.91/h$). Optimal overcapacity $\phi_{\mathrm{R0}}^{\mathrm{opt}}$ as a function of feast time T for different values of the initial and final nutrient qualities. (**f-j**) Absolute fitness $W(T) = M(T)/M(0)$ (mass accumulation) for the same three strains. (**k-o**) The contours show, for each strain with overcapacity ϕ_{R0}, and for each feast time T, the fitness $W(\phi_{\mathrm{R0}}, T)$ normalized by the optimal value $W^{\mathrm{opt}}(T) = W(\phi_{\mathrm{R0}}^{\mathrm{opt}}(T), T)$: $W/W^{\mathrm{opt}} > 90\%$ (white), $50\% < W/W^{\mathrm{opt}} < 90\%$ (light brown), $W/W^{\mathrm{opt}} < 50\%$ (dark brown). The three horizontal lines correspond to the three strains in the previous panels.

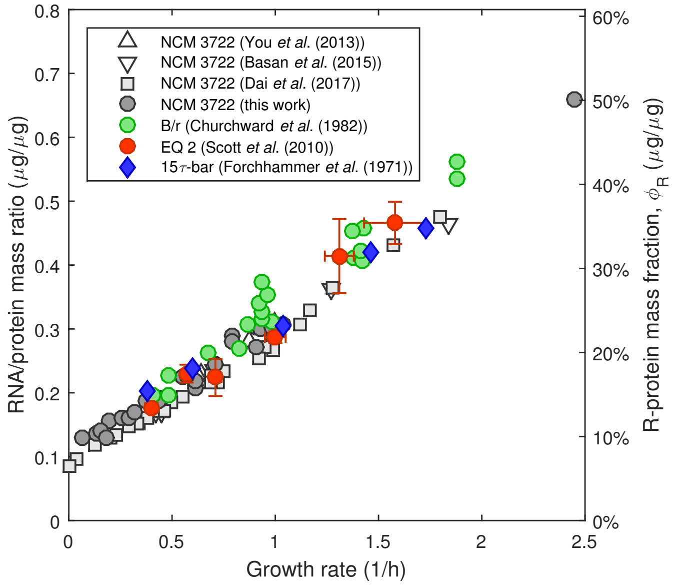

Supplementary Figure 6: RNA to protein mass ratio for different *E. coli* strains. In this figure we show the ratio of total RNA mass to total protein mass for different strains of *E. coli*, in exponential growth in different nutrient sources. The strains are: NCM 3722, in white or grey ([11],[9],[6]); EQ-2 (derived from MG 1655), red circles [4]; B/r, green circles [12]); 15τ-bar (an auxotroph, non K-12, strain), blue diamonds [10]. We show on the right axis the corresponding R-protein mass fraction, using the same conversion factor (0.76 grams of R-proteins per gram of RNA).

Symbol and definition	Description
M	Total protein mass of the cell population.
M_{R}	Mass of R-proteins, proportional to the mass of ribosomal proteins.
$\phi_{\mathrm{R}} = M_{\mathrm{R}}/M$	Mass fraction of R-proteins
$\dot{M} = \mathrm{d}M/\mathrm{d}t$	Protein synthesis rate
$\lambda = \dot{M}/M$	Growth rate
$\sigma = \dot{M}/M_R = \lambda/\phi_{\mathrm{R}}$	Translational efficiency
$\chi_{\mathrm{R}} = \dot{M}_R/\dot{M}$	Production rate of ribosomal proteins
λ_{i}, λ_{f}	Initial (pre-shift) and final (asymptotic post-shift) steady state growth rates
λ_0	Growth rate reached immediately after the nutritional shift.
$\Delta\lambda = \lambda_0 - \lambda_{\mathrm{i}}$	Jump in growth rate at the upshift.
$\phi^*_{\mathrm{R}} = \phi_{\mathrm{R0}} + \lambda^*/\nu_{\mathrm{R}}$	R-sector growth law, valid at steady state (indicated by the asterisk). The first term, ϕ_{R0}, is the "R-sector overcapacity" while the inverse of the slope (ν_{R}) matches the maximum translation capacity.
$\lambda_{\mathrm{R0}} = \phi_{\mathrm{R0}}\nu_{\mathrm{R}}$	"R-sector offset". It equals the maximum jump $\Delta\lambda$; in particular, $\Delta\lambda = \lambda_{\mathrm{R0}}$ when λ^*_{f} is much larger than λ_{R0} itself.

Supplementary Table 1: Symbols used in the text. This table lists all symbols used in the Main Text. Asterisks always indicate steady state, time-independent, quantities; when discussing nutritional shift, we use the apices/pedices i and f instead of the asterisks to indicate the (steady state) pre-shift or post-shift conditions, respectively. Similarly, the superscript WT refers to steady state quantities for a wild type strain; for example, λ^{WT} is the steady state growth rate of the wild type strain, and $\phi^{\mathrm{WT}}_{\mathrm{R0}}$ its R-protein overcapacity.

Pre-shift C-substrate	λ_i (1/h)	λ_0 (1/h)
0.2% Glucose	0.91 ± 0.03	1.04 ± 0.10
0.2% Arabinose	0.87 ± 0.03	0.99 ± 0.10
0.2% Glycerol	0.68 ± 0.03	0.86 ± 0.09
0.2% Fructose	0.63 ± 0.03	0.77 ± 0.08
0.2% Mannose	0.44 ± 0.03	0.72 ± 0.08
0.1% Mannose	0.36 ± 0.03	0.64 ± 0.08
0.075% Mannose	0.34 ± 0.03	0.68 ± 0.08
0.05% Mannose	0.30 ± 0.03	0.66 ± 0.07
20mM Glutamate	0.10 ± 0.03	0.50 ± 0.06
20mM Aspartate	0.06 ± 0.03	0.52 ± 0.07

Supplementary Table 2: Fit results. Fit results for the growth kinetics in various upshift experiments (these data are shown in Main Text Fig. 2c and Fig. 2d). Pre-shift growth rates λ_i^* are computed from the pre-shift growth curves or from separate steady state experiments; we estimate for them an error of 0.03/h. The values of λ_0 were extracted by fitting (the log-transformed) Eq. (7) from the main text to the experimental $log(M(t)/M(0))$ (data in Fig. S3). In each fit, λ_0 is the only fitting parameter; λ_f was instead set to the value $\lambda_\mathrm{f} = 2.45$/h, obtained for steady state exponential growth on LB + 0.2% glucose medium. Uncertainties for λ_0 arise from two sources. (1) The error arising from the scatter of $M(t)/M(0)$ can be estimated with a jack-knife resampling, yielding an error σ_jack (2) The error arising from the uncertainty in λ_f, $\sigma_{\lambda_\mathrm{f}} = 0.2/h$, can be propagated on λ_0 by numerically computing the derivative $\mathrm{d}\lambda_0/\mathrm{d}\lambda_\mathrm{f}$. The values reported here are computed by summing in quadrature the two errors, i.e. $\sigma^2_{\lambda_0} = \sigma^2_\mathrm{jack} + (\mathrm{d}\lambda_0/\mathrm{d}\lambda_\mathrm{f})^2\sigma^2_{\lambda_\mathrm{f}}$.

Supplementary References

[1] Schleif, R. (1967). Control of production of ribosomal protein. Journal of molecular biology, 27(1), 41-55.

[2] Dennis, P. P., & Bremer, H. (1974). Differential rate of ribosomal protein synthesis in Escherichia coli B/r. Journal of Theoretical Biology, 84(3), 407-422

[3] Bremer, H., & Dennis, P. P. (1975). Transition period following a nutritional shift-up in the bacterium Escherichia coli B/r: stable RNA and protein synthesis. Journal of theoretical biology, 52(2), 365-382.

[4] Scott, M., Gunderson, C. W., Mateescu, E. M., Zhang, Z., & Hwa, T. (2010). Interdependence of cell growth and gene expression: origins and consequences. Science, 330(6007), 1099-1102.

[5] Klumpp, S., Scott, M., Pedersen, M., and Hwa, T. (2013). Molecular crowding limits translation and cell growth. PNAS 110 (42) 1675416759

[6] Dai, X., Zhu, M., et al. (2016). Reduction of translating ribosomes enables Escherichia coli to maintain elongation rates during slow growth. Nature Microbiology, 2, 16231.

[7] Schaechter, M., Maaløe, O. and Kjeldgaard, O. (1958). Dependency on Medium and Temperature of Cell Size and Chemical Composition during Balanced Growth of Salmonella typhimurium. J. gen. Microbial. 19, 592-606

[8] Bremer, H., & Dennis, P. P. (1996). Escherichia coli and Salmonella: cellular and molecular biology. Washington (DC): American Society for Microbiology. Chapter, Modulation of chemical composition and other parameters of the cell by growth rate, 1553-1569.

[9] Basan, M., Zhu, M., Dai, X., Warren, M., Svin, D., Wang, Y. P., & Hwa, T. (2015). Inflating bacterial cells by increased protein synthesis. Molecular systems biology, 11(10), 836.

[10] Forchhammer, J., & Lindahl, L. (1971). Growth rate of polypeptide chains as a function of the cell growth rate in a mutant of Escherichia coli 15. Journal of molecular biology, 55(3), 563-568.

[11] You, C., et al. (2013). Coordination of bacterial proteome with metabolism by cyclic AMP signalling. Nature, 500(7462), 301-306.

[12] Churchward, G., Bremer, H., & Young, R. (1982). Transcription in bacteria at different DNA concentrations. Journal of bacteriology, 150(2), 572-581.
